# μ-Biphenyl-3,3′,4,4′-tetra­carboxyl­ato-κ^2^ *O* ^3^:*O* ^3′^-bis­[triaqua­(2,2′-bipyridyl-κ^2^ *N*,*N*′)nickel(II)] hexa­hydrate

**DOI:** 10.1107/S1600536809014639

**Published:** 2009-04-25

**Authors:** Dong Zhou, Min Shao, Xiang He, Yongmei Zhao, Shourong Zhu

**Affiliations:** a Department of Chemistry, Shanghai University, Shanghai 200444, People’s Republic of China; b Instrumental Analysis Center, Shanghai University, Shanghai 200444, People’s Republic of China

## Abstract

The asymmetric unit of the title complex, [Ni_2_(C_16_H_6_O_8_)(C_10_H_8_N_2_)_2_(H_2_O)_6_]·6H_2_O, contains one Ni^II^ atom, one 2,2′-bipyridine ligand, three coordinated water mol­ecules, one-half of a fully deprotonated biphenyl-3,3′,4,4′-tetra­carboxyl­ate anion and three lattice water mol­ecules. The Ni^II^ atom displays a distorted NiN_2_O_4_ octa­hedral coordination formed by one carboxyl­ate O atom, three water O atoms and two N atoms of the chelating ligand. The complete biphenyl-3,3′,4,4′-tetra­carboxyl­ate ligand displays inversion symmetry and links two symmetry-related Ni^II^ atoms into a binuclear complex. Neighbouring complex mol­ecules are linked through O—H⋯O hydrogen bonds into a three-dimensional structure. Additional O—H⋯O hydrogen bonds between the lattice water mol­ecules help to consolidate the crystal packing.

## Related literature

For other metal complexes with biphenyl-3,3′,4,4′-tetra­carboxyl­ate as ligand, see: Hao *et al.* (2005 ▶); Wang *et al.* (2005 ▶, 2006 ▶, 2007 ▶). For related structures containing biphenyl-3,3′,4,4′-tetra­carboxyl­ate and neutral chelating ligands, see: Zhu *et al.* (2008*a* ▶,*b* ▶).
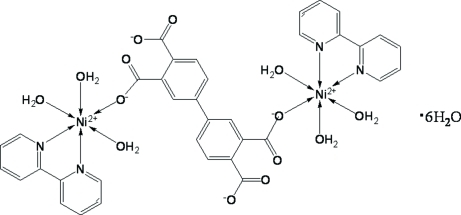


## Experimental

### 

#### Crystal data

[Ni_2_(C_16_H_6_O_8_)(C_10_H_8_N_2_)_2_(H_2_O)_6_]·6H_2_O
*M* *_r_* = 972.19
Triclinic, 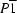

*a* = 7.5126 (14) Å
*b* = 12.088 (2) Å
*c* = 12.285 (2) Å
α = 105.445 (2)°
β = 98.075 (2)°
γ = 92.162 (3)°
*V* = 1061.4 (3) Å^3^
*Z* = 1
Mo *K*α radiation
μ = 0.97 mm^−1^
*T* = 296 K
0.20 × 0.20 × 0.15 mm

#### Data collection

Bruker SMART CCD area-detector diffractometer
Absorption correction: multi-scan (*SADABS*; Bruker, 2002 ▶) *T*_min_ = 0.829, *T*_max_ = 0.868
5556 measured reflections
3698 independent reflections
2526 reflections with *I* > 2σ(*I*)
*R*_int_ = 0.037

#### Refinement

*R*[*F* ^2^ > 2σ(*F* ^2^)] = 0.062
*wR*(*F* ^2^) = 0.172
*S* = 1.05
3698 reflections
280 parameters
H-atom parameters constrained
Δρ_max_ = 0.61 e Å^−3^
Δρ_min_ = −0.53 e Å^−3^

### 

Data collection: *SMART* (Bruker, 2002 ▶); cell refinement: *SAINT* (Bruker, 2002 ▶); data reduction: *SAINT*; program(s) used to solve structure: *SHELXS97* (Sheldrick, 2008 ▶); program(s) used to refine structure: *SHELXL97* (Sheldrick, 2008 ▶); molecular graphics: *DIAMOND* (Brandenburg & Putz, 2006 ▶) and *ORTEP-3 for Windows* (Farrugia, 1997 ▶); software used to prepare material for publication: *SHELXTL* (Sheldrick, 2008 ▶).

## Supplementary Material

Crystal structure: contains datablocks global, I. DOI: 10.1107/S1600536809014639/wm2225sup1.cif

Structure factors: contains datablocks I. DOI: 10.1107/S1600536809014639/wm2225Isup2.hkl

Additional supplementary materials:  crystallographic information; 3D view; checkCIF report

## Figures and Tables

**Table 1:** Selected bond lengths (Å)

Ni1—N2	2.063 (4)
Ni1—N1	2.064 (4)
Ni1—O2*W*	2.067 (3)
Ni1—O1	2.069 (3)
Ni1—O3*W*	2.075 (3)
Ni1—O1*W*	2.076 (4)

**Table 2:** Hydrogen-bond geometry (Å, °)

*D*—H⋯*A*	*D*—H	H⋯*A*	*D*⋯*A*	*D*—H⋯*A*
O1*W*—H1*WA*⋯O4^i^	0.82	1.91	2.720 (5)	168
O1*W*—H1*WB*⋯O3	0.85	2.04	2.889 (5)	176
O2*W*—H2*WA*⋯O2	0.82	1.99	2.708 (5)	146
O2*W*—H2*WB*⋯O3^ii^	0.84	2.06	2.715 (5)	135
O3*W*—H3*WA*⋯O3^i^	0.85	1.88	2.723 (5)	168
O3*W*—H3*WB*⋯O4*W*	0.82	1.97	2.793 (6)	178
O4*W*—H4*WA*⋯O2^iii^	0.87	1.87	2.715 (6)	164
O4*W*—H4*WB*⋯O5*W*	0.85	2.22	2.803 (8)	125
O5*W*—H5*WB*⋯O6*W*^iv^	0.83	2.18	2.770 (15)	128
O6*W*—H6*WA*⋯O2	0.85	2.44	3.091 (11)	134
O6*W*—H6*WA*⋯O2	0.85	2.44	3.091 (11)	134

Symmetry codes: (i) 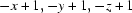; (ii) 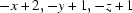; (iii) 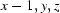; (iv) 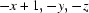.

**Fig. 1.**
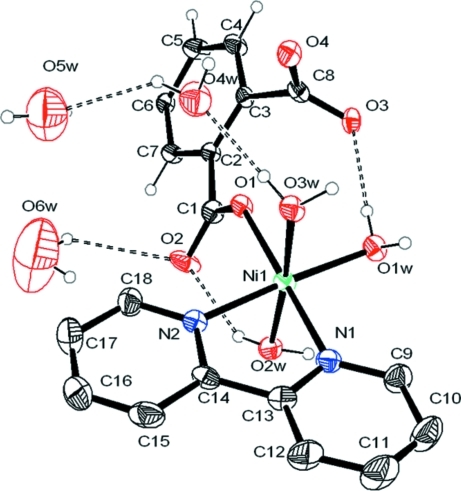
The asymmetric unit in the structure of complex (I), displayed with ellipsoids at the 50% probability level. Dashed lines represent hydrogen bonds.

**Fig. 2.**
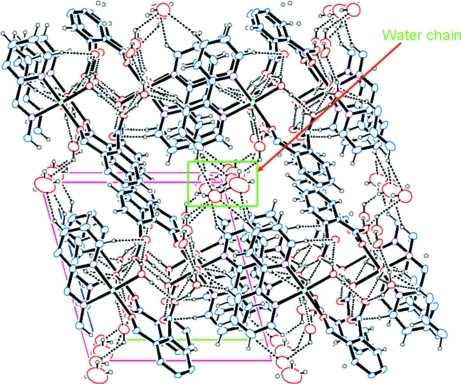
Crystal packing in the crystal structure of compound (I) viewed down the a axis with ellipsoids ath the 30% probability level.

**Fig. 3.**
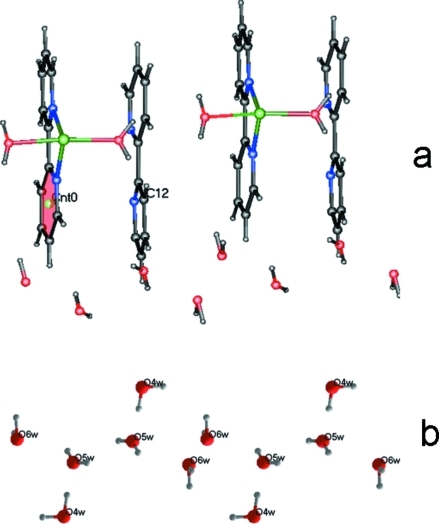
(a) The π—π interaction between adjacent 2, 2'-bipyridine molecules. (b) The crystal lattice water molecules arranged in chains via H -bonds; other atoms are omitted for clarity.

## supplementary crystallographic information

### Comment

Coordination compounds of biphenyl-3,3',4,4'-tetracarboxylic acid have been investigated previously. As expected, the deprotonated ligand coordinates to metal ions in a versatile mode due to its multidentate character (Hao *et al.*, 2005; Wang *et al.*, 2005; 2006; 2007). Upon adding chelating ligands, such as 2,2'-bipyridine or 1,10-phenanthroline, ternary coordination polymers can be formed (Zhu *et al.*, 2008a). In all these complexes, biphenyl-3,3',4,4'-tetracarboxylate acts as counter ion and/or multidentate ligand. Here we present the crystal structure of the dinuclear complex [Ni_2_(C_10_H_8_N_2_)_2_(C_16_H_6_O_8_)(H_2_O)_6_]^.^6H_2_O, (I).

The unique Ni atom in the structure of complex (I) (Fig. 1) is in a distorted octahedral coordination sphere formed by one carboxylate O, three water O and two N atoms with Ni—O and Ni—N bond lengths in the range 2.063 (4) Å - 2.076 (4) Å with σ=0.87 (Zhu *et al.*, 2008b). The fully deprotonated biphenyl-3,3',4,4'-tetracarboxylate ligand displays inversion symmetry and links two symmetry-related Ni^II^ atoms. Due to symmetric reason, the two benzene rings of the biphenyl ligand are coplanar. The two pyridine rings in the 2,2'-bipyridine molecule have a torsion angle of 4.7 (8)°. The carboxylate group that coordinate to nickel is almost coplanar with the benzene ring (torsion angle 8.6 (8)°), while the free carboxylate has a torsion angle of 72.2 (7)° with the benzene ring which is almost perpendicular each other. The intramolecular distance between the two nickel(II) ions is 14.788 (11) Å.

As expected, there are considerable hydrogen bonds in the structure. Table 2 lists bond distances and angles. These H-bonds link dinuclear complex together to a three-dimensional structure (Fig 2.). The uncoordinated crystal lattice water molecules interact through additional hydrogen bonds, as shown in Fig. 3, and thus help to consolidate the crystal packing.

### Experimental

A mixture of biphenyl-3,3',4,4'-tetracarboxylic acid dianhydride (0.5 mmol), 2,2'-bipyridine (0.5 mmol), NaOH (2 mmol) and Ni(NO_3_)_2_ (1 mmol) in 8 ml H_2_O was placed in a 25 ml Teflon reactor, which was sealed and heated in a oven at 433 K for 72 h. Then the autoclave was cooled to room temperature at the rate of 10 K to get light-blue flat crystals of the title compound (in *ca* 85% yield based on biphenyltetracarboxylic dianhydride). The crystals were isolated by filtration and washed with water.

### Refinement

The aromatic H atoms were generated geometrically and were included in the refinement in the riding model approximation (*d*(C—H) = 0.93 Å, *U*_iso_=1.2Ueq(C)). The H atoms of the water molecules were identified in difference Fourier syntheses and were refined with distance restraints of *d*(O—H) = 0.85 Å.

### Crystal data

**Table:**

[Ni_2_(C_16_H_6_O_8_)(C_10_H_8_N_2_)_2_(H_2_O)_6_]·6H_2_O	*Z* = 1
*M**_r_* = 972.19	*F*(000) = 506
Triclinic, *P*1	*D*_x_ = 1.521 Mg m^−^^3^
Hall symbol: -P 1	Mo *K*α radiation, λ = 0.71073 Å
*a* = 7.5126 (14) Å	Cell parameters from 1042 reflections
*b* = 12.088 (2) Å	θ = 2.8–21.2°
*c* = 12.285 (2) Å	µ = 0.97 mm^−^^1^
α = 105.445 (2)°	*T* = 296 K
β = 98.075 (2)°	Flat, light-blue
γ = 92.162 (3)°	0.20 × 0.20 × 0.15 mm
*V* = 1061.4 (3) Å^3^	

### Data collection

**Table:**

Bruker SMART CCD area-detector diffractometer	3698 independent reflections
Radiation source: fine-focus sealed tube	2526 reflections with *I* > 2σ(*I*)
graphite	*R*_int_ = 0.037
φ and ω scans	θ_max_ = 25.1°, θ_min_ = 2.1°
Absorption correction: multi-scan (*SADABS*; Bruker, 2002)	*h* = −8→8
*T*_min_ = 0.829, *T*_max_ = 0.868	*k* = −11→14
5556 measured reflections	*l* = −14→14

### Refinement

**Table:**

Refinement on *F*^2^	Primary atom site location: structure-invariant direct methods
Least-squares matrix: full	Secondary atom site location: difference Fourier map
*R*[*F*^2^ > 2σ(*F*^2^)] = 0.062	Hydrogen site location: inferred from neighbouring sites
*wR*(*F*^2^) = 0.172	H-atom parameters constrained
*S* = 1.05	*w* = 1/[σ^2^(*F*_o_^2^) + (0.0835*P*)^2^ + 0.0076*P*]
3698 reflections	(Δ/σ)_max_ < 0.001
280 parameters	Δρ_max_ = 0.61 e Å^−^^3^
0 restraints	Δρ_min_ = −0.53 e Å^−^^3^

### Special details

**Table:**

Geometry. All e.s.d.'s (except the e.s.d. in the dihedral angle between two l.s. planes) are estimated using the full covariance matrix. The cell e.s.d.'s are taken into account individually in the estimation of e.s.d.'s in distances, angles and torsion angles; correlations between e.s.d.'s in cell parameters are only used when they are defined by crystal symmetry. An approximate (isotropic) treatment of cell e.s.d.'s is used for estimating e.s.d.'s involving l.s. planes.
Refinement. Refinement of *F*^2^ against ALL reflections. The weighted *R*-factor *wR* and goodness of fit *S* are based on *F*^2^, conventional *R*-factors *R* are based on *F*, with *F* set to zero for negative *F*^2^. The threshold expression of *F*^2^ > σ(*F*^2^) is used only for calculating *R*-factors(gt) *etc*. and is not relevant to the choice of reflections for refinement. *R*-factors based on *F*^2^ are statistically about twice as large as those based on *F*, and *R*- factors based on ALL data will be even larger.

### Fractional atomic coordinates and isotropic or equivalent isotropic displacement parameters (Å^2^)

**Table:**

	*x*	*y*	*z*	*U*_iso_*/*U*_eq_	
Ni1	0.76324 (9)	0.27456 (6)	0.46907 (5)	0.0292 (2)	
C1	0.8729 (8)	0.3451 (5)	0.2678 (4)	0.0353 (13)	
C2	0.8455 (7)	0.4289 (4)	0.1965 (4)	0.0279 (12)	
C3	0.7072 (7)	0.5022 (4)	0.2062 (4)	0.0271 (11)	
C4	0.6778 (8)	0.5682 (5)	0.1309 (5)	0.0390 (14)	
H4	0.5816	0.6146	0.1345	0.047*	
C5	0.7894 (8)	0.5668 (5)	0.0497 (5)	0.0439 (16)	
H5	0.7662	0.6122	−0.0002	0.053*	
C6	0.9365 (7)	0.4985 (4)	0.0411 (4)	0.0294 (12)	
C7	0.9582 (7)	0.4283 (4)	0.1148 (4)	0.0325 (13)	
H7	1.0511	0.3793	0.1095	0.039*	
C8	0.5935 (8)	0.5223 (4)	0.3010 (5)	0.0319 (13)	
C9	0.7786 (9)	0.2720 (6)	0.7187 (5)	0.0498 (16)	
H9	0.7915	0.3519	0.7363	0.060*	
C10	0.7713 (11)	0.2212 (7)	0.8067 (6)	0.071 (2)	
H10	0.7805	0.2658	0.8820	0.086*	
C11	0.7506 (12)	0.1055 (7)	0.7801 (7)	0.078 (2)	
H11	0.7430	0.0692	0.8374	0.093*	
C12	0.7407 (10)	0.0414 (6)	0.6699 (6)	0.064 (2)	
H12	0.7271	−0.0385	0.6516	0.077*	
C13	0.7509 (7)	0.0962 (5)	0.5854 (5)	0.0384 (14)	
C14	0.7468 (7)	0.0348 (5)	0.4639 (5)	0.0362 (13)	
C15	0.7461 (9)	−0.0842 (5)	0.4239 (6)	0.0596 (19)	
H15	0.7454	−0.1292	0.4745	0.072*	
C16	0.7463 (11)	−0.1351 (6)	0.3104 (7)	0.075 (2)	
H16	0.7468	−0.2147	0.2837	0.091*	
C17	0.7460 (10)	−0.0692 (6)	0.2371 (6)	0.070 (2)	
H17	0.7472	−0.1023	0.1597	0.084*	
C18	0.7437 (8)	0.0492 (5)	0.2803 (5)	0.0490 (16)	
H18	0.7395	0.0946	0.2297	0.059*	
N1	0.7680 (6)	0.2112 (4)	0.6097 (4)	0.0375 (11)	
N2	0.7472 (6)	0.1002 (4)	0.3901 (4)	0.0333 (10)	
O1	0.7508 (5)	0.3349 (3)	0.3258 (3)	0.0327 (9)	
O2	1.0074 (6)	0.2870 (4)	0.2630 (4)	0.0519 (12)	
O3	0.6703 (5)	0.5796 (3)	0.3995 (3)	0.0357 (9)	
O4	0.4305 (5)	0.4876 (3)	0.2757 (3)	0.0414 (10)	
O1W	0.7793 (5)	0.4475 (3)	0.5584 (3)	0.0378 (9)	
H1WA	0.7075	0.4576	0.6039	0.045*	
H1WB	0.7528	0.4864	0.5108	0.045*	
O2W	1.0407 (5)	0.2754 (3)	0.4820 (3)	0.0366 (9)	
H2WA	1.0698	0.2647	0.4183	0.044*	
H2WB	1.0810	0.3312	0.5383	0.044*	
O3W	0.4835 (5)	0.2653 (3)	0.4440 (3)	0.0372 (9)	
H3WA	0.4238	0.3141	0.4852	0.045*	
H3WB	0.4455	0.2638	0.3776	0.045*	
O4W	0.3480 (6)	0.2550 (4)	0.2175 (4)	0.0662 (13)	
H4WA	0.2365	0.2698	0.2201	0.079*	
H4WB	0.3472	0.2363	0.1454	0.079*	
O5W	0.3527 (11)	0.0578 (6)	0.0358 (6)	0.141 (3)	
H5WA	0.4255	0.1162	0.0371	0.169*	
H5WB	0.3141	−0.0088	−0.0025	0.169*	
O6W	0.9624 (19)	0.0848 (9)	0.0429 (9)	0.288 (7)	
H6WA	0.9844	0.1042	0.1156	0.345*	
H6WB	0.9808	0.1578	0.0631	0.345*	

### Atomic displacement parameters (Å^2^)

**Table:**

	*U*^11^	*U*^22^	*U*^33^	*U*^12^	*U*^13^	*U*^23^
Ni1	0.0322 (4)	0.0294 (4)	0.0321 (4)	0.0051 (3)	0.0127 (3)	0.0145 (3)
C1	0.038 (3)	0.042 (3)	0.033 (3)	0.006 (3)	0.016 (3)	0.015 (3)
C2	0.028 (3)	0.033 (3)	0.027 (3)	0.006 (2)	0.012 (2)	0.012 (2)
C3	0.030 (3)	0.028 (3)	0.026 (3)	0.004 (2)	0.014 (2)	0.008 (2)
C4	0.038 (3)	0.048 (4)	0.041 (3)	0.019 (3)	0.023 (3)	0.019 (3)
C5	0.047 (4)	0.056 (4)	0.047 (4)	0.016 (3)	0.022 (3)	0.036 (3)
C6	0.034 (3)	0.036 (3)	0.023 (3)	0.007 (2)	0.015 (2)	0.012 (2)
C7	0.038 (3)	0.037 (3)	0.032 (3)	0.013 (3)	0.017 (2)	0.019 (2)
C8	0.038 (3)	0.027 (3)	0.038 (3)	0.009 (2)	0.018 (3)	0.015 (2)
C9	0.061 (4)	0.049 (4)	0.042 (4)	0.002 (3)	0.011 (3)	0.017 (3)
C10	0.098 (6)	0.083 (6)	0.038 (4)	0.000 (5)	0.012 (4)	0.025 (4)
C11	0.118 (7)	0.074 (6)	0.056 (5)	−0.004 (5)	0.015 (5)	0.044 (4)
C12	0.091 (6)	0.059 (5)	0.055 (5)	0.005 (4)	0.014 (4)	0.038 (4)
C13	0.034 (3)	0.039 (3)	0.046 (4)	0.000 (3)	0.004 (3)	0.018 (3)
C14	0.029 (3)	0.029 (3)	0.053 (4)	0.002 (2)	0.007 (3)	0.016 (3)
C15	0.066 (5)	0.041 (4)	0.077 (5)	0.003 (3)	−0.003 (4)	0.031 (4)
C16	0.111 (7)	0.037 (4)	0.069 (5)	0.010 (4)	0.000 (5)	0.005 (4)
C17	0.086 (6)	0.057 (5)	0.058 (5)	0.007 (4)	0.017 (4)	−0.003 (4)
C18	0.063 (4)	0.046 (4)	0.038 (4)	0.009 (3)	0.012 (3)	0.009 (3)
N1	0.038 (3)	0.043 (3)	0.038 (3)	0.011 (2)	0.012 (2)	0.017 (2)
N2	0.031 (3)	0.034 (3)	0.037 (3)	0.004 (2)	0.008 (2)	0.013 (2)
O1	0.037 (2)	0.038 (2)	0.032 (2)	0.0107 (17)	0.0208 (17)	0.0162 (17)
O2	0.051 (3)	0.065 (3)	0.065 (3)	0.036 (2)	0.039 (2)	0.042 (2)
O3	0.038 (2)	0.039 (2)	0.029 (2)	0.0016 (18)	0.0134 (17)	0.0021 (17)
O4	0.031 (2)	0.054 (3)	0.039 (2)	0.0020 (19)	0.0156 (18)	0.0096 (19)
O1W	0.043 (2)	0.039 (2)	0.040 (2)	0.0053 (18)	0.0209 (18)	0.0157 (18)
O2W	0.033 (2)	0.044 (2)	0.036 (2)	0.0058 (18)	0.0107 (17)	0.0136 (18)
O3W	0.033 (2)	0.045 (2)	0.039 (2)	0.0084 (18)	0.0172 (18)	0.0134 (18)
O4W	0.048 (3)	0.075 (3)	0.071 (3)	0.005 (2)	0.015 (2)	0.009 (3)
O5W	0.189 (8)	0.116 (6)	0.113 (6)	0.008 (5)	0.038 (5)	0.019 (5)
O6W	0.42 (2)	0.237 (14)	0.165 (11)	−0.049 (13)	0.054 (12)	−0.011 (9)

### Geometric parameters (Å, °)

**Table:**

Ni1—N2	2.063 (4)	C11—H11	0.9300
Ni1—N1	2.064 (4)	C12—C13	1.380 (8)
Ni1—O2W	2.067 (3)	C12—H12	0.9300
Ni1—O1	2.069 (3)	C13—N1	1.340 (7)
Ni1—O3W	2.075 (3)	C13—C14	1.475 (8)
Ni1—O1W	2.076 (4)	C14—N2	1.352 (7)
C1—O2	1.252 (6)	C14—C15	1.390 (8)
C1—O1	1.259 (6)	C15—C16	1.366 (9)
C1—C2	1.508 (7)	C15—H15	0.9300
C2—C3	1.388 (7)	C16—C17	1.351 (10)
C2—C7	1.400 (6)	C16—H16	0.9300
C3—C4	1.375 (7)	C17—C18	1.390 (8)
C3—C8	1.513 (7)	C17—H17	0.9300
C4—C5	1.387 (7)	C18—N2	1.322 (7)
C4—H4	0.9300	C18—H18	0.9300
C5—C6	1.403 (7)	O1W—H1WA	0.8201
C5—H5	0.9300	O1W—H1WB	0.8498
C6—C7	1.394 (7)	O2W—H2WA	0.8201
C6—C6^i^	1.489 (9)	O2W—H2WB	0.8379
C7—H7	0.9300	O3W—H3WA	0.8542
C8—O4	1.248 (6)	O3W—H3WB	0.8200
C8—O3	1.266 (6)	O4W—H4WA	0.8664
C9—N1	1.334 (7)	O4W—H4WB	0.8528
C9—C10	1.384 (8)	O5W—H5WA	0.8722
C9—H9	0.9300	O5W—H5WB	0.8339
C10—C11	1.347 (9)	O6W—H6WA	0.8500
C10—H10	0.9300	O6W—H6WB	0.8500
C11—C12	1.359 (10)		
			
N2—Ni1—N1	80.00 (17)	C9—C10—H10	120.9
N2—Ni1—O2W	88.41 (16)	C10—C11—C12	120.3 (6)
N1—Ni1—O2W	91.05 (16)	C10—C11—H11	119.9
N2—Ni1—O1	98.95 (15)	C12—C11—H11	119.9
N1—Ni1—O1	178.17 (17)	C11—C12—C13	119.4 (7)
O2W—Ni1—O1	90.43 (14)	C11—C12—H12	120.3
N2—Ni1—O3W	88.32 (16)	C13—C12—H12	120.3
N1—Ni1—O3W	91.12 (16)	N1—C13—C12	121.3 (6)
O2W—Ni1—O3W	175.71 (14)	N1—C13—C14	115.0 (5)
O1—Ni1—O3W	87.34 (14)	C12—C13—C14	123.7 (6)
N2—Ni1—O1W	176.34 (15)	N2—C14—C15	119.8 (6)
N1—Ni1—O1W	96.33 (16)	N2—C14—C13	116.8 (5)
O2W—Ni1—O1W	91.76 (15)	C15—C14—C13	123.4 (5)
O1—Ni1—O1W	84.71 (14)	C16—C15—C14	120.2 (6)
O3W—Ni1—O1W	91.67 (14)	C16—C15—H15	119.9
O2—C1—O1	123.8 (5)	C14—C15—H15	119.9
O2—C1—C2	119.8 (4)	C17—C16—C15	119.6 (7)
O1—C1—C2	116.3 (5)	C17—C16—H16	120.2
C3—C2—C7	119.6 (4)	C15—C16—H16	120.2
C3—C2—C1	121.8 (4)	C16—C17—C18	118.4 (7)
C7—C2—C1	118.6 (4)	C16—C17—H17	120.8
C4—C3—C2	119.0 (4)	C18—C17—H17	120.8
C4—C3—C8	116.6 (4)	N2—C18—C17	122.8 (6)
C2—C3—C8	124.2 (4)	N2—C18—H18	118.6
C3—C4—C5	121.1 (5)	C17—C18—H18	118.6
C3—C4—H4	119.5	C9—N1—C13	118.1 (5)
C5—C4—H4	119.5	C9—N1—Ni1	127.2 (4)
C4—C5—C6	121.6 (5)	C13—N1—Ni1	114.7 (4)
C4—C5—H5	119.2	C18—N2—C14	119.1 (5)
C6—C5—H5	119.2	C18—N2—Ni1	127.5 (4)
C7—C6—C5	116.1 (4)	C14—N2—Ni1	113.3 (4)
C7—C6—C6^i^	121.8 (6)	C1—O1—Ni1	129.2 (3)
C5—C6—C6^i^	122.1 (6)	Ni1—O1W—H1WA	109.6
C6—C7—C2	122.5 (5)	Ni1—O1W—H1WB	108.5
C6—C7—H7	118.8	H1WA—O1W—H1WB	109.4
C2—C7—H7	118.8	Ni1—O2W—H2WA	109.7
O4—C8—O3	125.0 (5)	Ni1—O2W—H2WB	105.3
O4—C8—C3	118.2 (5)	H2WA—O2W—H2WB	124.6
O3—C8—C3	116.7 (5)	Ni1—O3W—H3WA	122.7
N1—C9—C10	122.8 (6)	Ni1—O3W—H3WB	109.6
N1—C9—H9	118.6	H3WA—O3W—H3WB	105.8
C10—C9—H9	118.6	H4WA—O4W—H4WB	100.3
C11—C10—C9	118.1 (7)	H5WA—O5W—H5WB	143.0
C11—C10—H10	120.9	H6WA—O6W—H6WB	74.3

Symmetry codes: (i) −*x*+2, −*y*+1, −*z*.

### Hydrogen-bond geometry (Å, °)

**Table:**

*D*—H···*A*	*D*—H	H···*A*	*D*···*A*	*D*—H···*A*
O1W—H1WA···O4^ii^	0.82	1.91	2.720 (5)	168
O1W—H1WB···O3	0.85	2.04	2.889 (5)	176
O2W—H2WA···O2	0.82	1.99	2.708 (5)	146
O2W—H2WB···O3^iii^	0.84	2.06	2.715 (5)	135
O3W—H3WA···O3^ii^	0.85	1.88	2.723 (5)	168
O3W—H3WB···O4W	0.82	1.97	2.793 (6)	178
O4W—H4WA···O2^iv^	0.87	1.87	2.715 (6)	164
O4W—H4WB···O5W	0.85	2.22	2.803 (8)	125
O5W—H5WB···O6W^v^	0.83	2.18	2.770 (15)	128
O6W—H6WA···O2	0.85	2.44	3.091 (11)	134
O6W—H6WA···O2	0.85	2.44	3.091 (11)	134

Symmetry codes: (ii) −*x*+1, −*y*+1, −*z*+1; (iii) −*x*+2, −*y*+1, −*z*+1; (iv) *x*−1, *y*, *z*; (v) −*x*+1, −*y*, −*z*.
